# Au–ZnO Conjugated Black Phosphorus as a Near-Infrared Light-Triggering and Recurrence-Suppressing Nanoantibiotic Platform against *Staphylococcus aureus*

**DOI:** 10.3390/pharmaceutics13010052

**Published:** 2021-01-02

**Authors:** Atanu Naskar, Sohee Lee, Kwang-sun Kim

**Affiliations:** Department of Chemistry and Chemistry Institute for Functional Materials, Pusan National University, Busan 46241, Korea; atanunaskar@pusan.ac.kr (A.N.); kin5497170@pusan.ac.kr (S.L.)

**Keywords:** low-temperature synthesis, black phosphorus, antibacterial activity, photothermal therapy, drug resistance

## Abstract

Antibiotic therapy is the gold standard for bacterial infections treatment. However, the rapid increase in multidrug-resistant (MDR) bacterial infections and its recent use for secondary bacterial infections in many COVID-19 patients has considerably weakened its treatment efficacy. These shortcomings motivated researchers to develop new antibacterial materials, such as nanoparticle-based antibacterial platform with the ability to increase the chances of killing MDR strains and prevent their drug resistance. Herein, we report a new black phosphorus (BP)-based non-damaging near-infrared light-responsive platform conjugated with ZnO and Au nanoparticles as a synergistic antibacterial agent against *Staphylococcus aureus* species. First, BP nanosheets containing Au nanoparticles were assembled in situ with the ZnO nanoparticles prepared by a low-temperature solution synthesis method. Subsequently, the antibacterial activities of the resulting Au–ZnO–BP nanocomposite against the non-resistant, methicillin-resistant, and erythromycin-resistant *S. aureus* species were determined, after its photothermal efficacy was assessed. The synthesized nanocomposite exhibited excellent anti-*S. aureus* activity and good photothermal characteristics. The non-resistant *S. aureus* species did not produce drug-resistant bacteria after the treatment of multiple consecutive passages under the pressure of the proposed nanoantibiotic, but rapidly developed resistance to erythromycin. This work clearly demonstrates the excellent photothermal antibacterial properties of Au–ZnO–BP nanocomposite against the MDR *S. aureus* species.

## 1. Introduction

Infections caused by pathogenic bacteria seriously threaten human health; however, they can be effectively treated by antibiotics, which have served as the primary therapeutic weapon against bacterial infections for many decades. However, the abuse of these antibiotics paved the way for the emergence of multidrug-resistant (MDR) bacterial strains, rendering antibiotic treatments ineffective [1]. Bacterial resistance against antibiotics represents a significant healthcare issue. The prevalence of such bacterial infections can be illustrated by the projected death toll of 10 million people per year by 2050 [2]. One of the well known and dangerous MDR bacteria (listed among “Serious threats” in the report issued by the Centers for Decease Control and Prevention in 2019) [3] is methicillin-resistant *Staphylococcus aureus* (MRSA) that cannot be effectively treated by the majority of commercially available antibiotics.

In some cases, a recurrence of staphylococcal infections was observed after the treatment, owing to their ability to remain in the dormant state within human cells for months or even years [4]. Another worrying fact is the occurrence of secondary bacterial infections in many COVID-19 patients during the current global pandemic [5]. Almost all COVID-19 patients have been treated with antibiotics to prevent secondary bacterial infections despite their inability to kill the virus itself [6]. Therefore, the absence of an efficient COVID-19 therapy combined with an increasing use of antibiotics can produce a strong damaging effect on human health and a global growth of the antibiotic-resistant bacterial pathogens with worse outcomes. The treatment of these bacterial infections will become more challenging, owing to the increasing gap between the development of new effective antibiotics and the generation of new varieties of MDR bacterial species [1]. In a recent news on the relation between COVID-19 and MDR, it is reported that the MRSA is one of the seven most common MDR species infecting COVID-19 patients [7]. Therefore, it is imperative to consider various alternatives to antibiotics for curing a silent epidemic of MDR bacterial infections specially by MRSA.

Recently, two-dimensional (2D) biomedical nanomaterials including graphene, titanium carbide, carbon nitride (C_3_N_4_), boron nitride (BN), and black phosphorus (BP) have received significant attention from researchers [8]. Among these materials, BP is considered a “rising star” in the biomedical field, owing to its superior physiochemical properties as compared with those of other 2D materials [9]. Importantly, BP can serve as a support and an in situ green reductant for metal precursors (Ag and Au) unlike other 2D nanomaterials [10,11]. In addition, the high surface area of BP facilitates the loading of multiple antibacterial agents with broad absorption ranges across the ultraviolet, visible, and infrared spectra for biomedical applications [10]. Notably, the near-infrared (NIR) light can penetrate tissues with little side effects and therefore be effectively utilized in photothermal therapy [12], especially in the treatment methods involving BP, owing to its excellent NIR absorption properties.

Although the NIR light-mediated photothermal therapy is generally applied against cancer [13], it is also considered a promising alternative antibacterial strategy, in which the light energy is converted to thermal energy under NIR irradiation (700–1400 nm) and therefore helps inhibit the growth of bacterial cells [14]. In this regard, BP can act as an excellent NIR light-responsive photothermal antibacterial agent, owing to its strong NIR light absorption properties. Additionally, BP nanosheets (NSs) with specific 2D structures are able to penetrate bacterial membranes, owing to the ‘‘nanoknife effect’’ [8]. These NSs can also react with multiple antibacterial agents to produce combinatorial molecular nanoplatforms with synergistically enhanced antibacterial activities [10,11]. Therefore, the unique physiochemical properties of BP make it an ideal nanoplatform candidate material for biomedical applications, including antibacterial methods. However, despite the promising results reported by Liang et al. [15], Zhang et al. [16], Aksoy et al. [17], Zhang et al. [18], and Wu et al. [10], regarding the antibacterial activities of BP nanomaterials, the issue of drug resistance has not been properly addressed to date. Additionally, ZnO (ZO), a well known antibacterial agent [19], has not been conjugated with BP to synergistically increase its antibacterial activity.

Among inorganic nanomaterials, Au [1], Ag [20,21,22], and ZO [1] nanoparticles (NPs) have been widely used as antibacterial agents. ZO exhibits not only excellent antibacterial properties, but a remarkable ability to inhibit the recurrence of drug resistance by bacteria, as shown in our previous study [23]. Furthermore, although Au NPs represent the major catalytic and antibacterial components [10,24], they must be pre-treated with ligands to increase their stability, which often affect the intrinsic physicochemical characteristic and subsequent activity of these materials. Therefore, it would be beneficial to develop Au NPs without compromising their essential properties or employing any surfactants. One possible way of achieving this goal is to utilize BP as a green reductant during the synthesis. Additionally, Au NPs can show absorption bands ranging from the visible to the near-infrared (NIR) regions that have resulted in photothermal activity irradiated by NIR light [17]. Therefore, this optical property of Au NPs can also be exploited.

Considering the excellent antibacterial and drug resistant properties of Au and ZO NPs as well as the photothermal properties of Au NPs and BP NS, we prepared a new NIR light-assisted synergistic Au–ZO–BP (AZB) nanoplatform and evaluated its antibacterial activity against *S. aureus* species under NIR irradiation and the possible emergence of bacterial resistance. Consequently, strong suppressing activity against bacterial resistance was observed even after 10 treatment passages under the proposed nanoantibiotic pressure, whereas high resistance to erythromycin within several passages was rapidly developed by the same species. To the best of our knowledge, this is the first study describing an AZB nanoplatform with the NIR-assisted photothermal antibacterial activity that can potentially eradicate drug-resistant species.

## 2. Experimental

### 2.1. Synthesis of ZnO (ZO) NPs

The utilized ZO synthesis procedure was described in our previous work [23]. Initially, 50 mL of 0.2 M aqueous solution of zinc nitrate hexahydrate (Zn(NO_3_)_2_.6H_2_O, 98%, Sigma-Aldrich, St. Louis, MO, USA) in deionized water was prepared under continuous stirring for 15 min at 25 °C. In another beaker, 50 mL of 1.6 M aqueous solution of sodium hydroxide (NaOH, 95%, Junsei, Tokyo, Japan) was prepared by a similar procedure. Subsequently, the NaOH solution was added dropwise to the Zn(NO_3_)_2_ reaction mixture under continuous stirring to obtain a white precipitate at the bottom of the beaker. The mixture was then stirred at 80 °C for 6 h and transferred to an ice bath to stop the reaction. The obtained white precipitate was collected by centrifugation, washed with deionized water and ethanol several times, and dried inside an air oven at 60 °C overnight. It is worthy to note that the synthesis process is easily reproducible.

### 2.2. Syntheses of BP NS, ZO–BP Nanocomposite, and AZB Nanocomposite

#### 2.2.1. Synthesis of BP NSs

BP NSs were prepared from bulk BP crystals by the ultrasonication-assisted aqueous exfoliation of bulk BP according to a previously developed method [11]. First, 2.0 g of NaOH was added to 60 mL of N-methyl-2-pyrrolidone (NMP) and sonicated in a water bath for 5 min. The supernatant was collected by centrifugation. Subsequently, 25 mg of bulk BP crystals (99.998% purity, SKU:03933, Smart-elements GmbH, Vienna, Austria) were added to the NMP-containing saturated NaOH solution, and the resulting suspension was ultrasonicated by an ice bath ultrasonicator for 8 h at a bath temperature below 20 °C. After the completion of the exfoliation step, the unexfoliated BP crystals were removed by centrifuging the dispersion at 2000 rpm for 15 min, while the BP NSs were collected from the supernatant by subsequent centrifugation at 13,000 rpm for 10 min. The produced BP NS were dispersed in water to obtain a brown solution that was stored at 4 °C until further use. 

#### 2.2.2. Syntheses of ZB and AZB Nanocomposites

Briefly, 10.0 mL of aqueous 0.01 M chloroauric acid (HAuCl_4_.3H_2_O, Au ≥ 49%) was gradually added to the prepared BP NSs (4 mL of 1.5 mg mL^−1^) in water under sonication. The obtained mixture was added to 200 mg of the as synthesized ZO in 30 mL of deionized water and ultrasonicated for 10 min followed by continuous stirring for 6 h. After the reaction completion, AZB nanocomposite particles were collected by centrifugation and vacuum drying at 60 °C for 6 h. It is noteworthy that no surfactants were used to reduce Au NPs as BP acted as a green reductant during this process. A similar procedure was employed for the synthesis of the ZB nanocomposite except for the use of chloroauric acid.

### 2.3. Characterization

#### 2.3.1. Material Properties

X-ray diffraction (XRD) patterns of the as prepared ZO, ZB, and AZB samples were acquired in the 2θ range of 5°–80° using an X-ray diffractometer (D8 Advance with the DAVINCI design, Bruker, Billerica, MA, USA) equipped with a nickel-filtered Cu Kα radiation source (λ = 1.5406 Å). Transmission electron microscopy (TEM; Bruker Nano GmbH, Berlin, Germany) was utilized to evaluate the microstructure of a representative AZB sample. The analyzed samples were held by carbon-coated 300 mesh Cu grids during observations. An Axis Supra scanning X-ray photoelectron spectroscopy (XPS) microprobe surface analysis system (Kratos Analytical, Manchester, UK) was used to determine the binding energies and chemical states of the elements in the representative AZB sample in the binding energy range from 200 to 1200 eV. The C 1s peak position at 284.5 eV was employed as the binding energy reference.

#### 2.3.2. Determination of Photothermal Effect

The as prepared ZO, ZB, and AZB samples were irradiated by a NIR light source (808 nm, MDL-III-808-2.5 W, Optoelectronics Tech. Co. Ltd., Jilin, China) with a power of 2.5 W at a distance of 5 cm. The same amount of deionized water (0.5 mL) was used as a control in the photothermal effect studies. A temperature increment was recorded every 60 s by an infrared thermometer (Cat. No. 830-T1, Testo, Ltd., Croydon, South Australia) for 5 min. The photothermal effects of the AZB nanocomposites with various concentrations (10–100 µg mL^–1^) were evaluated by a similar method.

#### 2.3.3. Preparation of Bacterial Cells

Antibacterial activities of the as prepared samples were examined by monitoring the bacterial growth via optical density measurements. Initially, *S. aureus* (ATCC 25923) (www.atcc.org) and clinical MRSA isolates [25] were prepared on a Luria–Bertani agar plate. Sample colonies were diluted to an optical density of 0.5 McFarland turbidity standard using a Sensititre™ nephelometer (Thermo Fisher Scientific, Waltham, MA, USA) followed by the inoculation (10^5^ CFU mL^–1^) into BBL^TM^ Mueller Hinton broth (MHB, BD Biosciences, Franklin Lakes, NJ, USA). After the addition of ZO, ZB, or AZB NPs, the cells were cultivated at 37 °C overnight under vigorous shaking to measure their antibacterial activity (Section 2.3.4), characterizing the cell morphology (Section 2.3.5), and assess the drug resistance (Section 2.3.6).

#### 2.3.4. Measurement of Antibacterial Activity

Proposed antibiotic samples with controls are divided into eight groups: (1) blank (control), (2) NIR, (3) ZO, (4) ZO + NIR, (5) ZB, (6) ZB + NIR, (7) AZB, and (8) AZB + NIR. To measure the antibacterial activity of the proposed antibiotics, the prepared bacterial cells of *S. aureus* ATCC 25923 and MRSA strains were seeded into a 96-well plate with the above categorized samples to a final concentration at 25 and 50 µg mL^−1^, respectively. In groups (2), (4), (6), and (8), cells were irradiated by the 808 nm laser with a power of 2.5 W at a 5 cm distance for 5 min before the cultivation. Bacterial growth for 20 h at 37 °C with 500 rpm was monitored by measuring the absorbance at a wavelength of 600 nm (OD_600_) using a spectrophotometric microplate reader (BMG LABTECH GmbH, Ortenberg, Germany).

#### 2.3.5. Morphological Characterization of Bacteria

The antibacterial effects of AZB against *S. aureus* and MRSA strains were examined. For this purpose, AZB (5 mg mL^−1^) was added to the cell suspension with the final concentration specified in Section 2.3.4. Both strains were prepared with (sample group) and without (control group) NIR irradiation and incubated at 37 °C overnight. The incubated cells were collected by centrifugation at 12,000 rpm for 1 min. The cells were then resuspended in 500 µL of a phosphate-buffered saline solution (pH = 7) containing 2% formaldehyde and 1% glutaraldehyde followed by centrifugation for 5 min. The obtained cell pellet was washed twice and resuspended in 1 mL of deionized water. A 5 µL aliquot was collected from the suspension and deposited on a silicon wafer (5 × 5 mm^2^, Namkang Hi-Tech Co., Ltd., Seongnam, Korea) to dry at room temperature. VEGA3, a versatile tungsten thermionic emission scanning electron microscopy (SEM) system (TESCAN, Fuveau, France), was used to study the dried wafer according to the protocol described by the manufacturer.

#### 2.3.6. Drug Resistance Assessment

The minimum lethal concentration (MLC) is defined as the required concentration of antibacterial material (antibiotics or nanocomposite) to reduce 99.9% initial inoculum of bacteria after incubation for 16 h in terms of OD_600_ values. Initially, the MLC for erythromycin and AZB nanocomposite was measured against *S. aureus* (Passage 1). Then, the occurrence of the drug resistance of AZB or erythromycin to the sub-lethal concentration (0.5 MLC) was monitored by incubating AZB or erythromycin to the cells from Passage 1 for further passages of growth. (Passages 2–10). The MLC of AZB and erythromycin against each passage of *S. aureus* species were evaluated to determine the relative occurrence of bacterial resistance to the AZB sample. Finally, the occurrence of resistance as fold changes of MLC was calculated using following equation: MLC_i+1_/_i_, where i is the passage of growth from 1 to 9.

## 3. Results and Discussion

### 3.1. Material Properties

#### 3.1.1. Phase Composition

**Figure 1:**
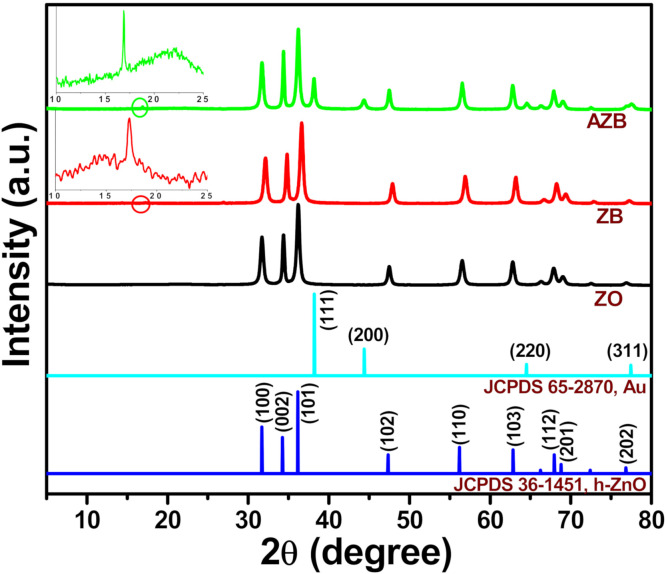
XRD patterns of the ZO, ZB, and Au–ZO– black phosphorus (BP) (AZB) samples. Insets illustrate the enlarged regions in the ZB and AZB spectra marked by the red circles.

The crystalline phases of the ZO, ZB, and AZB samples were determined from their XRD profiles as depicted in Figure 1. The XRD reflection peaks of the as synthesized ZO, ZB, and AZB centered at 31.62°, 34.27°, 36.21°, 47.41°, 56.50°, 62.70°, 67.81°, 68.97°, and 76.93° correspond to the (100), (002), (101), (102), (110), (103), (112), (201), and (202) diffraction planes, respectively, which are consistent with the hexagonal ZO (h-ZO) structure (Joint Committee on Powder Diffraction Standards (JCPDS) 36-1451) [23]. Moreover, some additional peaks were observed at approximately 38.05°, 44.25°, and 64.54° for the AZB sample, which contained the (111), (200), and (220) crystal planes of cubic Au NPs (JCPDS 65-2870) [26]. These results confirmed the successful formation of Au NPs without using any surfactants, during which BP acted as a green reductant. The enlarged spectra of the ZB and AZB samples contain an additional peak at 17.15°, which corresponds to the (020) lattice plane of BP [27]. Therefore, the results of the XRD measurements confirmed the successful preparation of the ZO, ZB, and AZB samples.

#### 3.1.2. Morphology and Microstructure

**Figure 2:**
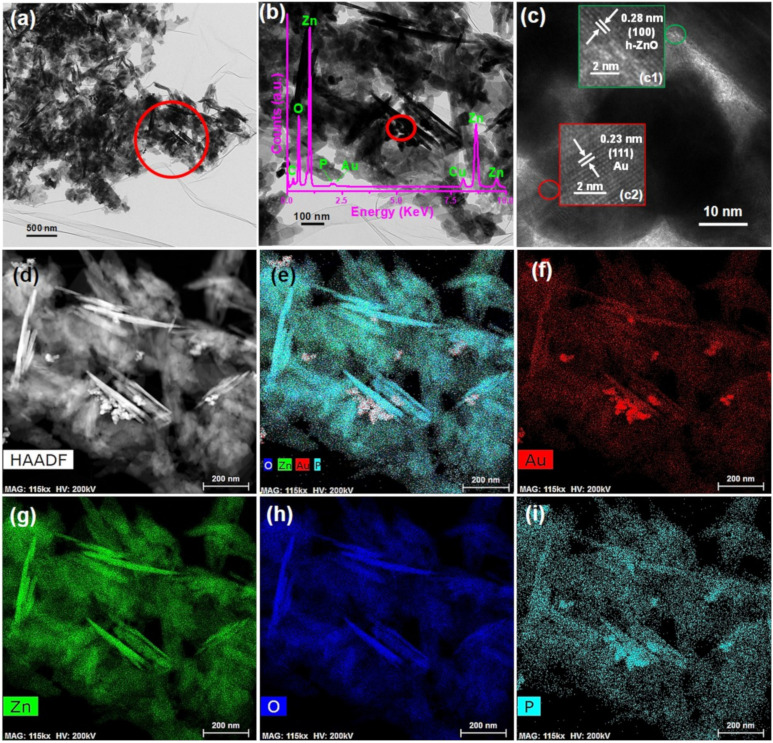
Characterization of the AZB sample. (**a**) TEM image of the AZB sample. (**b**) Enlarged TEM image of the marked region in the panel of (**a**). Inset displays the energy-dispersive X-ray spectrum of the AZB sample. (**c**) HRTEM image of the marked region in the panel of (**b**). Insets (c1) and (c2) display the HRTEM images of ZO and Au NPs, respectively. (**d**) High-angle annular dark-field (HAADF) image of the AZB sample and elemental maps of (**e**) the AZB composite, (**f**) Au, (**g**) Zn, (**h**) oxygen (O), and (**i**) phosphorus (P).

Figure 2 illustrates the morphology and microstructure of the representative AZB sample. The TEM images depicted in Figure 2a,b clearly indicate the formation of ZO NPs (Figure 2b displays the enlarged image of the marked region in Figure 2a). Similarly, Figure 2c illustrates the high-resolution TEM (HRTEM) image of the marked region in Figure 2b. This confirms the presence of Au and ZO NPs, as the distinct lattice fringes with interplanar distances of 0.23 and 0.28 nm depicted in Figure 2c correspond to the Au (111) [26] and ZO (100) [23] planes, respectively. Additionally, the inset of Figure 2b displays the energy-dispersive X-ray spectrum of the AZB sample. In this spectrum, the Zn and O peaks resulted from the formation of ZO NPs; the P peak originated from BP, and the Au peak was caused by the formation of Au NPs. The presence of C and Cu species can be attributed to the use of carbon-coated Cu grids during TEM measurements. Furthermore, the elemental mapping images of the representative AZB sample exhibit good distributions of Au (Figure 2f), Zn (Figure 2g), O (Figure 2h), and P (Figure 2i) elements. Therefore, the TEM and HRTEM images of the AZB sample confirmed the successful formation of Au and ZO NPs with BP NS, which were consistent with the XRD spectra (Figure 1).

#### 3.1.3. XPS Results

**Figure 3:**
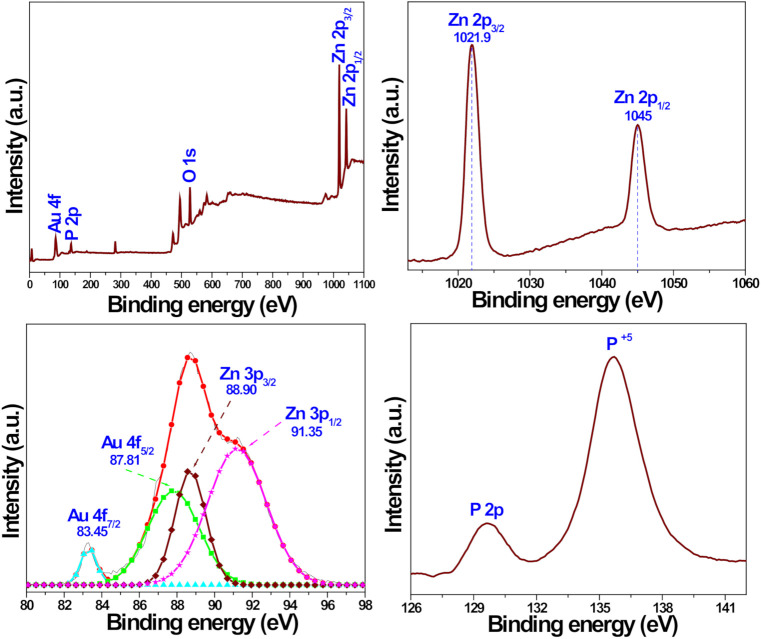
XPS spectra of the AZB nanocomposite. (**a**) Survey spectrum illustrating the presence of constituent elements in the analyzed sample. (**b**) Zn *2p* peaks. (**c**) Gaussian-fitted curves obtained for the Zn *3p* and Au *4f* peaks. (**d**) P *2p* core peaks.

Figure 3 illustrates the XPS spectra of the representative AZB sample, which were recorded to determine the surface chemical composition and valence state of the constituent elements. The XPS survey spectrum displayed in Figure 3a indicates the presence of Au, P, Zn, and O elements. The Zn *2p*, Au *4f*, and P *2p* peaks are shown in Figure 3b–d, respectively. The two relatively strong peaks centered at 1021.9 and 1045.0 eV in Figure 3b were assigned to the binding energies of the Zn *2p_3/2_* and Zn *2p*_1/2_ states, respectively [23]. Moreover, the calculated difference (~23.1 eV) between the Zn *2p_3/2_* and Zn *2p_1/2_* binding energies corresponded to the Zn^2+^ valence state [23], while Figure 3c confirmed the formation of metallic Au NPs in the AZB sample. In particular, its two peaks are centered at approximately 83.45 and 87.81 eV, which represent the binding energies of the Au *4f_7/2_* and Au *4f_5/2_* states, respectively [28]. However, the two other XPS peaks obtained by Gaussian fitting have binding energies of approximately 88.90 and 91.35 eV, which correspond to the Zn *3p_3/2_* and Zn *3p_1/2_* states, respectively [28]. Finally, the P *2p* peak is displayed in Figure 3d. Therefore, the obtained XPS data are in good agreement with the XRD (Figure 1) and TEM (Figure 2) results, which suggest the successful formation of the AZB nanocomposite.

### 3.2. Photothermal Effect

**Figure 4:**
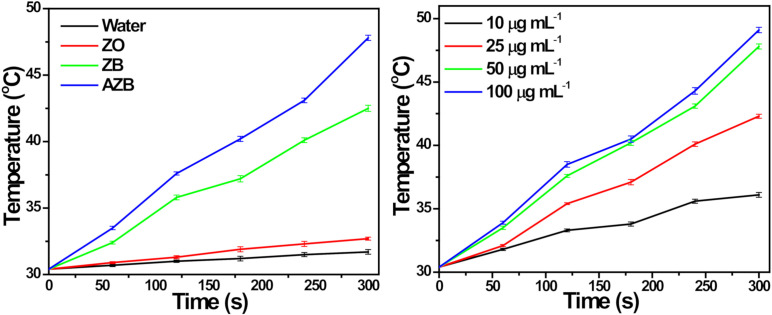
Photothermal temperature measurement. (**a**) Photothermal temperature increase observed for pure water and ZO, ZB, and AZB samples under NIR light irradiation. (**b**) Photothermal temperature increment observed for the AZB samples with different concentrations (the data are shown as the mean of triplicates).

The photothermal conversion of the AZB nanocomposite was explored under NIR light irradiation. Figure 4a illustrates the temperature increments observed for the ZO, ZB, and AZB samples with 50 µg mL^−1^ concentration and pure water. No temperature changes were detected in the absence of light irradiation, while the temperatures of the ZB and AZB samples increased by 12.1 and 17.4 °C, respectively, after 5 min of NIR light irradiation (Figure 4a). However, the temperature changes of pure water and the ZO sample were negligible under the same experimental conditions. Therefore, the temperature increments of the ZB and AZB samples are proportional to the photothermal conversion efficiency of BP NS [11]. Additionally, the photothermal effect of the AZB sample is strongly correlated with the AZB concentration, as shown in Figure 4b. The characteristic photothermal effect of BP NS was not affected by the synthesis of Au NPs in the AZB nanocomposite as the temperature of AZB exceeded that of the ZB nanocomposite. These results confirmed the effective conversion of the light energy into heat by BP NS through photoabsorption at 808 nm. 

### 3.3. Antibacterial Activity

**Figure 5:**
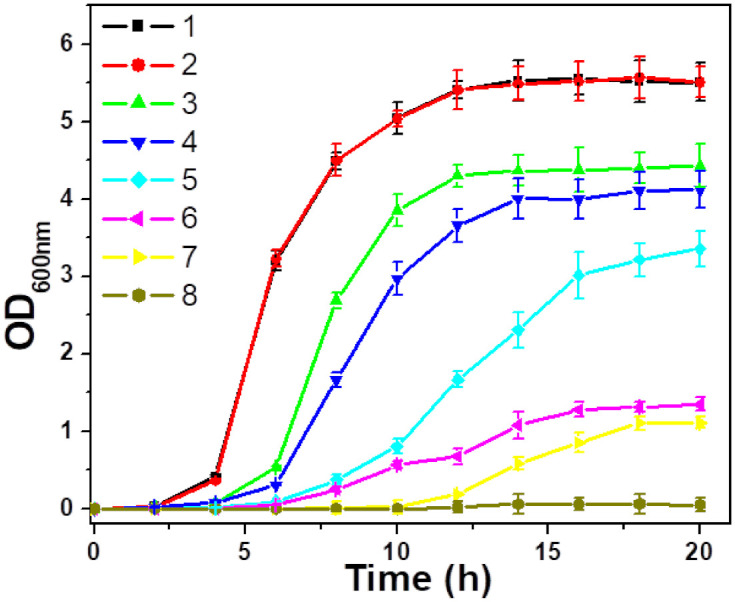
Growth curves of *S. aureus* species subjected to different treatments: (**1**) blank (control); (**2**) NIR; (**3**) ZO; (**4**) ZO + NIR; (**5**) ZB; (**6**) ZB + NIR; (**7**) AZB; and (**8**) AZB + NIR. The concentration of each nanomaterial (ZO, ZB, and AZB) was 25 μg mL^−1^ (the data are shown as the mean of triplicates).

**Figure 6:**
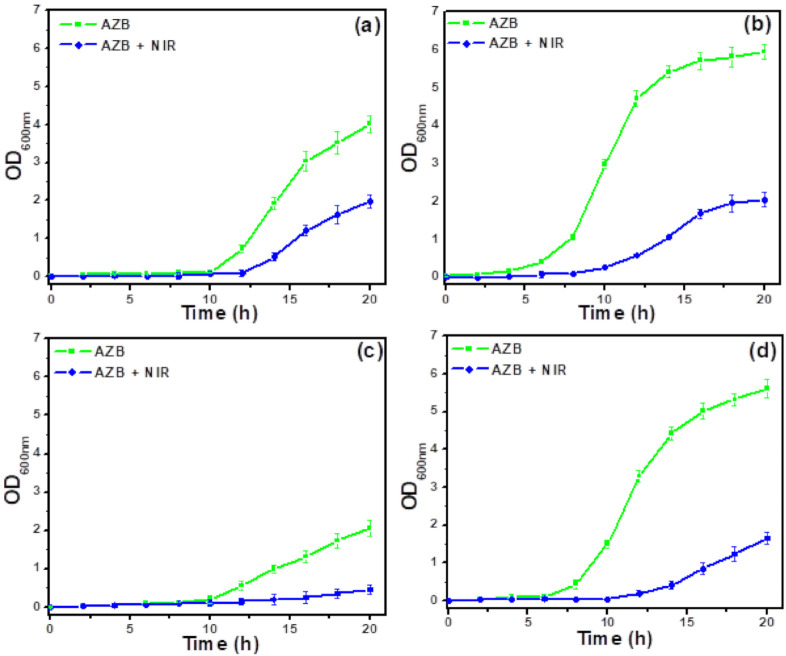

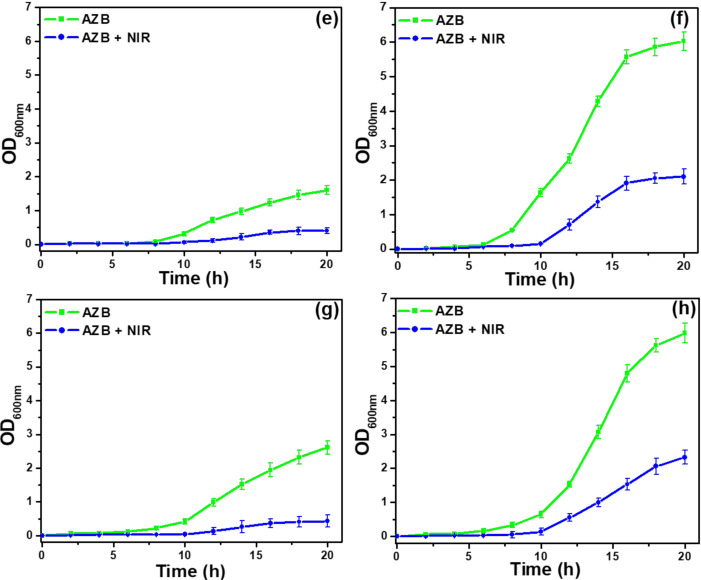
Effect of NIR on antibacterial activity of AZB. Growth curves of (**a**) MRSA1, (**b**) MRSA2, (**c**) MRSA3, (**d**) MRSA4, (**e**) MRSA5, (**f**) MRSA6, (**g**) MRSA7, and (**h**) MRSA8 cells subjected to the AZB and AZB + NIR treatments. The concentration of each AZB nanomaterial was 50 μg mL^−1^ (the data are shown as the mean of triplicates).

The antibacterial activities of the studied samples are evaluated by measuring the optical densities of the corresponding *S. aureus* and MRSA suspensions at 600 nm (OD_600_) in Figure 5 and Figure 6, respectively. According to Figure 5, the BP-conjugated samples (ZB and AZB) exhibited antibacterial activity against *S. aureus* species even without NIR light irradiation in contrast to the ZO sample, which could be attributed to the antibacterial properties of the ZO, BP, and Au [11,16,23]. However, after the initial inhibition, the ZB and AZB samples were unable to contain the bacterial growth. It is noteworthy that the AZB sample has managed to suppress the bacterial growth for up to 12 h, which exceeded the corresponding times obtained for the ZB and ZO samples, confirming the synergistic effect of these two components. Surprisingly, after irradiating the AZB sample, the bacterial growth was inhibited for a longer period (as compared with the time obtained for the other samples), and almost no bacterial growth was observed after 20 h. This phenomenon can be explained by the strong NIR light responsive photothermal property of BP [11] and Au [17] combined with the antibacterial properties of the ZO, BP, and Au. Therefore, the AZB sample was selected for further antibacterial studies. Figure 6a–h display the growth curves of eight MDR *S. aureus* strains (MRSA1–8) treated with the AZB nanomaterials that were obtained before and after NIR light irradiation. The strong activity of AZB enhanced its antibacterial properties toward all MRSA strains under NIR irradiation (as compared with those of non-irradiated AZB), which was similar to the data obtained for *S. aureus* species (Figure 5). Moreover, the ratio of the OD_600_ values at the end point of the antibacterial graph (i.e., 20 h) were 2.03 (MRSA1), 2.87 (MRSA2), 4.57 (MRSA3), 3.40 (MRSA4), 4.12 (MRSA5), 2.86 (MRSA6), 6.26 (MRSA7), and 2.55 (MRSA8) to the control sample. These results suggest the possibility of using AZB NPs as photothermal antibacterial agents against MDR *S. aureus* species.

### 3.4. Plausible Antibacterial Mechanism of the AZB Nanocomposite

**Figure 7:**
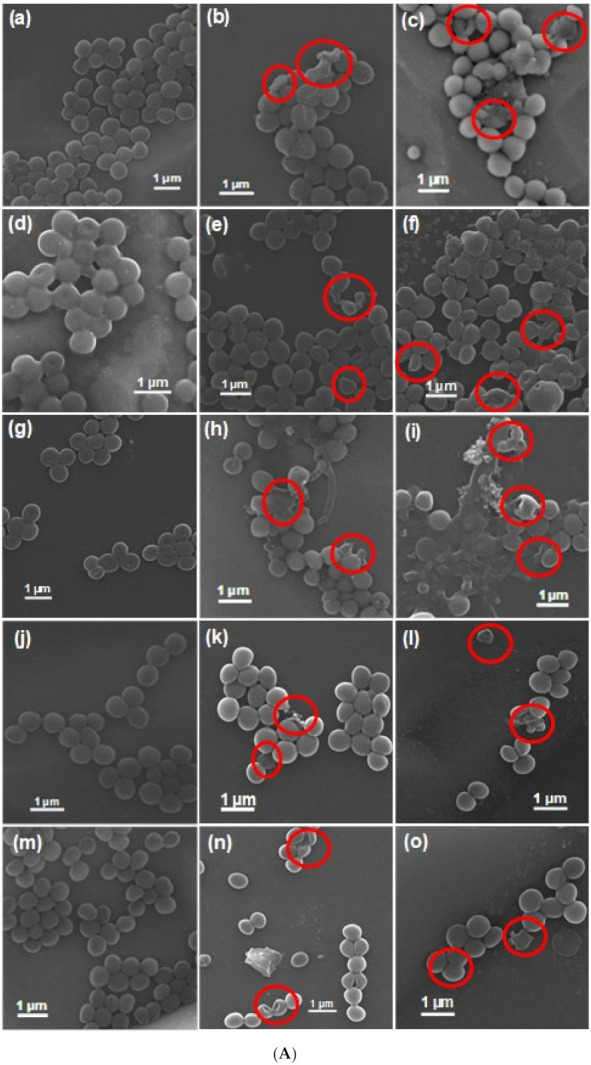

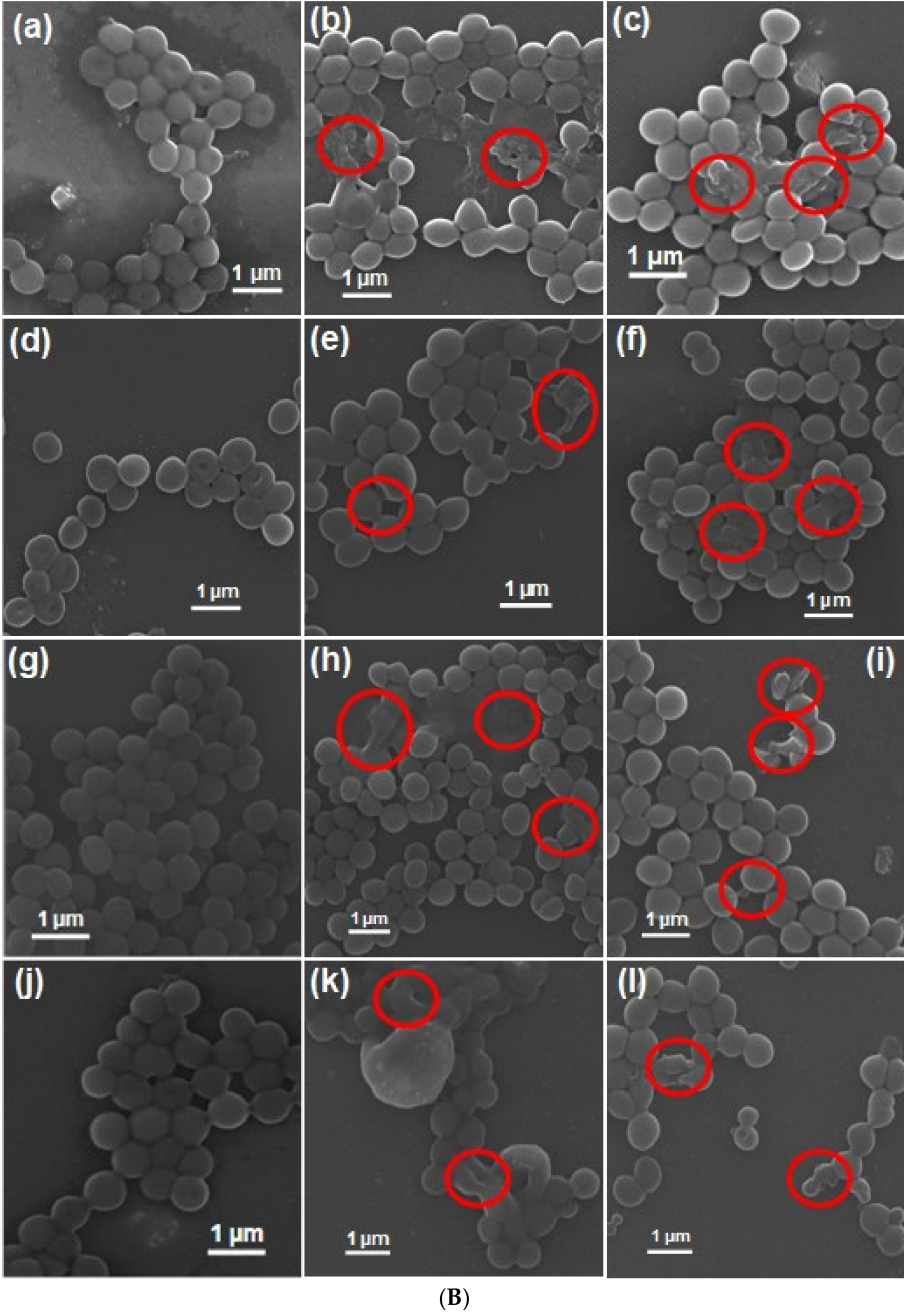
SEM images of bacterial cells after various treatments. (**A**) Untreated (a) *S. aureus*, (d) MRSA1, (g) MRSA2, (j) MRSA3, and (m) MRSA4. AZB-treated (b) *S. aureus*, (e) MRSA1, (h) MRSA2, (k) MRSA3, and (n) MRSA4. AZB + NIR-treated (c) *S. aureus*, (f) MRSA1, (i) MRSA2, (l) MRSA3, and (o) MRSA4. (**B**) Untreated (a) MRSA5, (d) MRSA6, (g) MRSA7, and (j) MRSA8. AZB-treated (b) MRSA5, (e) MRSA6, (h) MRSA7, and (k) MRSA8. AZB + NIR-treated (c) MRSA5, (f) MRSA6, (i) MRSA7, and (l) MRSA8. Disrupted cell membrane regions by treatments are indicated by the red circles.

To elucidate the plausible antibacterial mechanism of the NIR light-irradiated AZB nanocomposite and further verify its antibacterial properties, the morphological changes of the bacterial cells treated with AZB and AZB + NIR were investigated by SEM (Figure 7A,B). The obtained images clearly indicate that the untreated bacteria cells exhibit distinct spherical morphologies with smooth surfaces. However, the AZB-treated bacterial cells with and without NIR irradiation contain notably disrupted and wrinkled membranes with morphological defects, which inhibit the bacterial growth. It is noteworthy that the NIR-mediated AZB sample appeared to be significantly more effective than the non-irradiated AZB sample, confirming its high photothermal activity and the ability to inhibit bacterial growth.

### 3.5. Drug Resistance

**Figure 8:**
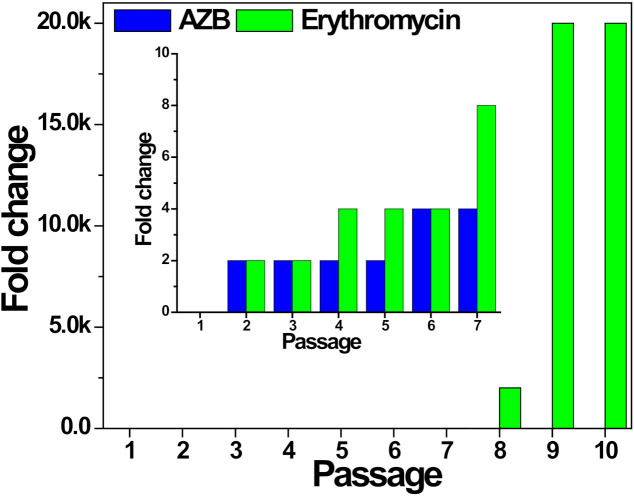
Acquisition of resistance by the treatment of AZB and erythromycin against *S. aureus* ATCC 25923 cells after multiple passages of growth. The fold change of minimum lethal concentration (MLC) (MLC_i+1_/_i_, where i is the passage of growth from 1 to 9) for AZB and erythromycin with the pressure of their sub-lethal concentration (0.5 MLC) was regarded as the indicator for the development of drug resistance. The inset displays the enlarged region including passages 1–7. Experiments were performed in triplicates and showed the same results in all experiments. The values are also indicated in Table 1.

The antibacterial activity of the AZB nanocomposite is strongly dependent on the photothermal membrane disruption; hence, it can be expected that the AZB efficiency is independent of the direct interactions of AZB with bacterial components, such as proteins and nucleic acids. Therefore, the chances of building bacterial resistance to the AZB nanocomposite are much less than those of cellular target-associated antibiotics. Therefore, erythromycin, a widely used antibiotic for the treatment of bacterial infections against *S. aureus,* was compared with the AZB sample in terms of drug resistance (the obtained results are shown in Figure 8). In this case, antibacterial activity in terms of minimum lethal concentration (MLC), which is defined in this study as the required concentration of antibacterial agents to reduce 99.9% initial inoculum of bacteria after incubation for 16 h in terms of OD_600_ values, and the corresponding fold change for *S. aureus* ATCC 25923 treated with AZB or erythromycin at sub-lethal concentrations (0.5 MLC) for 10 passages, was compared (Table 1). The fold change of MLC (MLC_i+1_/_i_, where i is the passage of growth from 1 to 9) was measured. The initial MLC for erythromycin and AZB against *S. aureus* were 0.25 and 50 µg mL^−1^, respectively. As shown in Figure 8, erythromycin was unable to escape an abrupt increase in the *S. aureus* resistance to the antibiotic pressure after increasing the number of passages and has ultimately lost its initial antibacterial activity. A significant increase (2000-fold change in 10 passages from intimal activity) was observed for erythromycin against *S. aureus*. Conversely, the AZB sample almost retained its initial value (4-fold change) to *S. aureus* even after 10 passages. A similar antibacterial effect of AZB was observed for MRSA. As indicated in Table 1, the antibacterial activity for the MRSA5 strain grown with 10 passages almost (2-fold change) retained its initial MLC value (100 µg mL^−1^). Moreover, the *S. aureus* cells with acquired erythromycin resistance (here named ERSA) exhibited similar activities (4-fold change after nine passages) as indicated by Table 1. Overall, the AZB sample demonstrated a relatively low risk of acquiring drug resistance after multiple exposures and therefore, represents a viable alternative for the long-term treatment of MDR *S. aureus* without recurrence. 

## 4. Conclusions

A new BP nanoplatform responsive to NIR light was constructed as a photothermal antibacterial agent for the treatment of MDR species. The synthesized nanocomposite was able to inhibit the growth of *S. aureus* species, owing to the intrinsic photothermal effect of BP NSs. BP NSs served as a green reductant during the synthesis of Au NPs for the AZB nanocomposite. The antibacterial activity of this nanocomposite was verified by its photothermal effect, bacterial growth curves, and SEM images. Moreover, the synthesized AZB nanocomposite exhibited very low bacterial resistance during its long-term usage, whereas the bacterial resistance to erythromycin increased rapidly within several passages of antibiotic pressures. This new BP-based nanoplatform, compared to other published reports, was not only synthesized firstly with Au and ZnO, but also showed excellent antibacterial activity along with very low bacterial resistance even throughout its continued use. Therefore, the proposed NIR-responsive nanoplatform can be potentially used as a new antibacterial agent and adjuvant against MDR *S. aureus* species.

## Figures and Tables

**Table 1:** Antibacterial activity comparison of (**a**) erythromycin (Ery) against *S. aureus* (SA) ATCC 25923 cells and the AZB samples against (**b**) SA (**c**) MRSA5, and (**d**) ERSA cells.

Passages	Fold Change			
	(a) Ery/SA	(b) AZB/SA	(c) AZB/MRSA5	(d) AZB/ERSA
1	N.D.	N.D.	N.D.	N.D.
2	2	2	2	N.D.
3	2	2	2	N.D.
4	4	2	2	N.D.
5	4	2	2	2
6	4	4	2	N.D.
7	8	4	2	N.D.
8	2000	4	2	4
9	20,000	4	2	4
10	20,000	4	2	N.D.

N.D.: not determined; the fold change of MLC (MLC_i+1_/_i_, where i is the passage of growth from 1 to 9) for AZB and erythromycin with the pressure of their sub-lethal concentration (0.5 MLC) was calculated. Experiments were performed in triplicates and showed the same results in all experiments.

## Data Availability

Not applicable.
